# Size at Birth, Postnatal Growth, and Reproductive Timing in an Australian Microbat

**DOI:** 10.1093/iob/obac030

**Published:** 2022-07-29

**Authors:** D L Eastick, S R Griffiths, J D L Yen, K A Robert

**Affiliations:** School of Agriculture, Biomedicine and Environment, La Trobe University, Melbourne, Victoria 3086; Centre for Future Landscapes, La Trobe University, Melbourne, Victoria 3086; School of Agriculture, Biomedicine and Environment, La Trobe University, Melbourne, Victoria 3086; Centre for Future Landscapes, La Trobe University, Melbourne, Victoria 3086; Arthur Rylah Institute for Environmental Research, Department of Environment, Land, Water and Planning, Heidelberg, Victoria 3084; School of Agriculture, Biomedicine and Environment, La Trobe University, Melbourne, Victoria 3086; Centre for Future Landscapes, La Trobe University, Melbourne, Victoria 3086

## Abstract

Reproductive phenology, size at birth, and postnatal growth are important life history traits that reflect parental investment. The ability to document detailed changes in these traits can be a valuable tool in the identification and management of at-risk wildlife populations. We examined reproductive traits in a common, widespread Australian microbat, *Chalinolobus gouldii*, at two sites over two years and derived growth curves and age estimation equations which will be useful in the study of how intrinsic and extrinsic factors alter parental investment strategies. We found that male and female offspring did not differ significantly in their size at birth or their postnatal growth rates. Bats born in 2018 were smaller at birth but grew at a faster rate than those born in 2017. When date of birth was compared across sites and years, we found bats born in 2018 had a later median birthdate (by 18 days) and births were more widespread than those born in 2017. Cooler and wetter weather during late gestation (Nov) in 2018 may have prolonged gestation and delayed births. With many bats facing threatening processes it is important to study reproductive plasticity in common and widespread “model” species, which may assist in the conservation and management of threatened microbats with similar reproductive traits.

## Introduction

Anthropogenic disturbance is a major threat to biodiversity (Jetz et al. 2007), with wildlife increasingly exposed to novel climates and land uses. Increased temperatures, altered rainfall patterns, and more frequent and extreme weather events (Cowan et al. 2014; Australian Bureau of Meteorology and CSIRO 2020) are already associated with adverse effects on biodiversity (Parmesan and Yohe 2003; Warren et al. 2013). Predicted changes in climate are expected to have increasingly severe impacts, including changes in species’ ranges, community structure, and ultimately, in ecosystem function (Bellard et al. 2012; Nunez et al. 2019).

Predicting the impacts of anthropogenic disturbance on wildlife is challenging without detailed knowledge of fitness-related traits, such as survivorship (van de Pol et al. 2010; Skagen and Adams 2012; Dybala et al. 2013), reproductive success (Adams 2010; Leblond et al. 2013; McHuron et al. 2017), and reproductive timing (Walther et al. 2002; Leech and Crick 2007; Love et al. 2010). In mammals, pregnancy and lactation are energetically demanding periods, especially in volant species (McLean and Speakman 1997), and anthropogenic impacts on reproductive success can negatively impact population growth (Cox et al. 2019; Pirotta et al. 2019).

For temperate-zone insectivorous bats (hereafter, bats), cold winters, and corresponding low insect availability can result in pregnancy and lactation being restricted to spring and summer (Kunz et al. 1998), with the fine-scale timing of these reproductive phases linked to year-to-year variation in climate cues (e.g., temperature and rainfall) (Frick et al. 2010). For example, climate can impact reproductive seasonality (variation in timing of reproductive events across two or more consecutive years) and reproductive synchrony (the temporal spread of birth dates within a population in a single year) in bats (Ransome and McOwat 1994; Frick et al. 2010; Eghbali and Sharifi 2019). Moreover, differing climate conditions across years have been found to influence gestation length (Willis et al. 2006), size at birth (Hoying and Kunz 1998; Hood et al. 2002), and postnatal growth rates in bats (McOwat and Andrews 1995; Dietz et al. 2007; Eghbali and Sharifi 2019). Reproductive phenology in bats is therefore likely to be sensitive to climate change (Jones and Rebelo 2013; Sherwin et al. 2013). Consequently, establishing baseline information on reproductive phenology and postnatal development will support attempts to monitor and predict the impacts of future climate change on bats. Early markers of reproductive success (e.g., reproductive phenology, size at birth, postnatal development) can be measured in free-ranging mammals and provide reliable proxies for postnatal survival or lifetime reproductive success (Kunz et al. 2009). Common, widespread bat taxa that occur across large geographic and climatic scales have potential to act as “model” species from which key findings can be transferred to less-common or threatened species where repeated measurements on individuals are difficult.

Only 5% of the > 1300 recognized species of bats worldwide have been studied for reproductive phenology, size at birth, and postnatal growth (Kunz et al. 2009; Fenton and Simmons 2015). Within Australia, two species of *Pteropus* bats have been studied for juvenile growth, *Pteropus poliocephalus* (Welbergen 2010) and captive *Pteropus conspicillatus* (Mclean et al. 2019), but to date no Australian microbats have been studied. This is partly due to the transient roosting patterns and inaccessible roosting sites of many bats, particularly tree-cavity roosting species. Artificial roosting boxes (bat boxes) can provide year-round access to bats, and a unique opportunity to document life history traits that are typically difficult to quantify in free-ranging species (Boyd and Stebbings 1989; Kerth and Reckardt 2003; Godinho et al. 2015; Lentini et al. 2015; Culina et al. 2017; Griffiths et al. 2017; Walker et al. 2020). Furthermore, use of bat boxes is typically dominated by common, widespread species (Mering and Chambers 2014; Griffiths et al. 2017).

Here, we conducted a fine-scale study on the reproductive phenology and postnatal development of free-ranging Gould's wattled bats (*Chalinolobus gouldii*), a common tree-roosting insectivorous species with a broad distribution encompassing most of the Australian continent (Australasian Bat Society 2021). We surveyed two discrete populations of *C. gouldii* that roost in bat boxes at two bushland reserves within the urban matrix of Greater Melbourne, Victoria, south-eastern Australia. The bats are part of a long-term mark-recapture study investigating various aspects of their ecology and life history (Irvine and Bender 1995; Bender 2011; Griffiths et al. 2017; Griffiths et al. 2019). We collected additional measurements on pups to provide further insight into *C. gouldii* reproduction and life history by describing the patterns in timing of birth, size at birth, and postnatal growth across two breeding seasons (2017–2018). We developed equations for estimating age during the linear growth period (first 20 days) and produced best-fit growth curves on postnatal growth patterns in body mass and forearm length using three non-linear growth equations (Logistic, Gomperts, and von Bertalanffy). Finally, we compared size at birth and postnatal growth across the 2017 and 2018 breeding seasons, and documented climate variables during both years.

## Methods

### Study species and sites

Gould's wattled bats (*C. gouldii*) are widespread across Australia, historically roosting in tree-hollows but also actively utilizing artificial roosts. Mating occurs in the austral autumn (Apr–May) resulting in sperm storage in the female reproductive tract over winter (Jun–Aug) with ovulation and fertilization occurring at the end of winter (Kitchener 1975). In south-eastern Australia, *C. gouldii* give birth annually during late spring to early summer (Oct–Dec) to twin pups (Churchill 2008), and occasionally biannually (second litter in late January/early February; DE and SG, *unpublished data*).

**Fig. 1:**
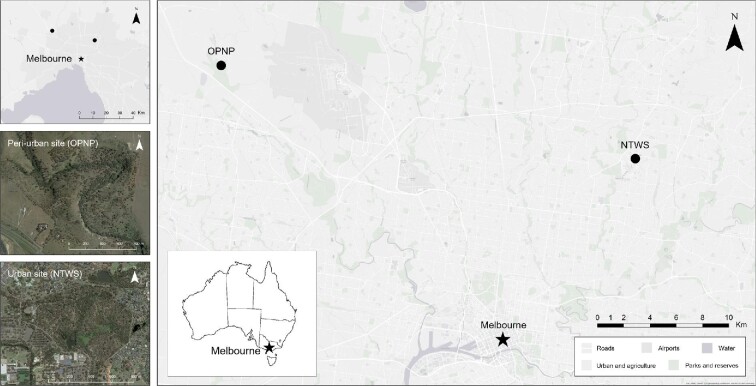
Location of the two reserves across greater Melbourne, Victoria. NTWS = Nangak Tamboree Wildlife Sanctuary, OPNP = Organ Pipes National Park.

This study examined *C. gouldii* using bat boxes at two bushland reserves in greater Melbourne (Fig. 1). Nangak Tamboree Wildlife Sanctuary (NTWS, formally La Trobe Wildlife Sanctuary) is a 30-ha river red gum (*Eucalyptus camaldulensis*) grassy woodland, 11 km north-east of the city center (37°39′55.58′′ S, 144°46′12.79′′ E). Organ Pipes National Park (OPNP) is a 152.5-ha site, located 35 km north-west of the Melbourne city center along the peri-urban border (37˚42′43.01′′ S, 145˚04′19.32′′ E). The predominant vegetation is river red gum interspersed with manna gum (*Eucalyptus viminalis*), with an understory of *Acacia* spp. and grasses. The site was revegetated in 1972 from heavily cleared farmland and consequently there are fewer hollow-bearing trees and a large uptake of artificial roosting boxes by *C. gouldii* (Griffiths et al. 2019) compared to the NTWS site.

### Data collection

We used longitudinal (mark–recapture) sampling on mothers and pups over two successive years (2017–2018). Bats were collected from boxes by hand during the day at regular intervals (averaging 3–4 days) during the first 60 days of the reproductive season and then every second month for 12 months, to encompass the reproductive period from parturition date throughout lactation, weaning, and independence. All bats within a box were placed into calico cloth bags until processing. Processing was done on-site to limit the time bats were kept out of their boxes. This protocol was employed to minimize the amount of disturbance to the mother and pups. Any female bat that was lactating was placed separately in a calico bag with her pups still attached and then following processing was returned to the roost box of collection.

Pups with the umbilical cord (and sometimes placenta) still attached were presumed to be born that day (Kunz 1973). Pups that did not have an umbilical cord attached but whose forearm length fell within one standard deviation (1SD) of the mean forearm length of pups captured with umbilicus attached were assumed to have been captured on day 1 (Hoying and Kunz 1998). Due to their small size and inability to carry arm bands until mature, on their first capture, all pups were identified by insertion of a microchip (Trovan Nano Transponder; Passive Integrated Transponder (PIT) tag), and the injection site sealed with tissue glue (3M Vetbond^TM^) (van Harten et al. 2021). A total of 475 *C. gouldii* pups (from 256 mums) were PIT-tagged across the two sites during 2017 and 2018 (NTWS 2017 = 151; OPNP 2017 = 4; NTWS 2018 = 67; OPNP 2018 = 110). Of the 475 pups PIT-tagged, 56 were captured on their day of birth (NTWS 2017 = 18; OPNP 2017 = 4; NTWS 2018 = 7; OPNP 2018 = 27), with 36 pups having an attached umbilical cord and 20 within 1SD of mean forearm length across both sites in 2017 and 2018. Pups that were not found on day 1 had their birth dates back calculated from the regression equation produced from known-age young (described below).

Each pup's sex was determined visually by the presence/absence of a penis. Forearm (FA) measurements were taken of the length of the right forearm from three repeated measurements to the nearest 0.1 mm using digital vernier calipers (Carbon Fiber Composites). While it has been suggested that forearm length may not be a good indicator of body size, it is the only measure currently appropriate for small bats (McGuire et al. 2018). The fourth metacarpal epiphyseal gap was also measured using calipers by spreading out the wing over a light box (transparent plastic sheet with illumination underneath). We attempted to perform all measurements on pups still attached to their mother, but if pups inadvertently became dislodged from the mother's nipple during this process, the pup was opportunistically weighed on digital scales to the nearest 0.1 g before reattaching to the nipple. Neonate (day of birth) weight was recorded for six pups (two female and four males) that were dislodged from the nipple. Other defining morphological features recorded included fur growth (none, fine “velvety” fur or fully furred), eyes (opened or closed), and skin pigmentation on the head and shoulders (light, medium, and dark). Once pups were fully furred and unattached from mother's nipple, the nipple was observed for signs of lactation (i.e., milk pooling below the skin).

All measurements of forearm length and epiphyseal gap throughout development were taken between November and March of 2017/2018 and 2018/2019, while body mass in bats that remained in the OPNP population was measured for the entire year following birth.

### Statistical analysis

We used a series of linear and non-linear models to assess differences in size-at-birth between sexes and years and in growth curves between years. We used a linear mixed-effects model (LMM) to compare the forearm lengths and epiphyseal gap lengths on day of birth between sexes and years (*n* = 56). As mass was collected from only six pups on day of birth, a model for mass was not produced. Most day of birth measurements collected in 2017 were from NTWS (*n* = 18 vs. *n* = 4 at OPNP) and most collected in 2018 were from OPNP (n = 27 vs. n = 7 at NTWS). Given this unbalanced sample collection, we used year of sampling as a predictor variable in all analyses but note that year and site are confounded. Both models included maternal ID as a random effect. All data were tested for normality using the Shapiro test, and unequal variances tested using Bartlett's test.

Growth data of the 56 young bats (forearm length and mass) were fitted to three non-linear growth models: the Gompertz equation, the logistic equation, and the von Bertalanffy equation (see Table 2 for equations). These growth models were fitted as non-linear mixed models with maternal ID as a random effect. In addition, growth models for forearm length were extended to test for differences in growth between years. The best fit for the data was determined by the Akaike's Information Criterion (AIC). There were insufficient mass data to fit to the von Bertalanffy model.

To develop age-predictive equations from forearm length, body mass, and epiphyseal gap, we fitted a LMM with age (in days) as the response variable and the size variable as the predictor (*n* = 56; Kunz and Anthony 1982). This model was additionally run with year as a predictor variable to compare forearm lengths between 2017 and 2018. We restricted this model to the linear portion of the age-growth association (forearm length: 1–21 days; body mass: 1–21 days; epiphyseal gap: 10–85 days) and included maternal ID as a random effect.

We used a generalized additive model (GAM) to describe non-linear changes in body mass of male and female *C. gouldii* pups throughout their first year of life. Only pups that we collected repeated measurements on were included in the model, and two female pups were removed from the data set as their weights were very low for female late-Autumn (*n* = 136). They were assumed to be very late born pups (February), not comparable with the November–December born pups included in this analysis.

Daily rainfall and half-hourly dry-bulb temperature readings were obtained from the Bureau of Meteorology (Australian Bureau of Meteorology 2021) for weather stations closest to each site (NTWS: #86,068; OPNP: #86,282). We used a LMM with three-way interactions between year, month, and site to determine if daily mean minimum and maximum temperatures for each month were different between sites and years.

All analyses were conducted in R version 4.0.3 (R Core Team 2020). We used the lmerTest R package to fit LMMs (Kuznetsova et al. 2017) and the nlme package to fit non-linear growth models (Pinheiro et al. 2021). We used the mgcv package to fit GAMs (Wood 2011). Confidence intervals (CI) for LMMs were calculated with the bootMER function in the lme4 R package (Bates et al. 2015).

## Results

### Reproductive ecology

Of the mothers captured with pups, 90% produced twins with parturition occurring between November and December in both years. At birth, neonates were naked with pink skin and their eyes closed (description of further developmental milestones are provided in Table 1). Pups were always found attached to the mother's nipple in roosts during the day until day 15, after this they became increasingly mobile in the roost and were intermittently attached to the mother. However, there were instances of lactating mothers roosting in boxes with no pups present, then several days later found with their dependent pups (approximately 13–14 days old) attached, a possible sign of temporary creche behavior. These occurrences became more frequent after day 25, therefore pups may be beginning to fly on their own at 3.5–4 weeks old. Swelling of the nipple and pooling of milk under the mother's skin was no longer visible between days 30–36 when pup forearm lengths were 96–98% of their adult length (calculated as a percentage of their individual adult forearm length; Table 1). This may be an indication that pups were now volant and beginning to forage on their own, but possibly still feeding on some milk from their mother until weight plateaued around day 45.

**Table 1:** Life history characteristics and growth parameters of juvenile *C. gouldii* monitored from day of birth (Day 1).

Life history, characteristics, and growth parameters	All data	2017	2018
*Neonates*			
*n*	56	22	34
Sex ratio (% males)	51.8	36.4	61.8
Mean mass at birth¹ (g)	2.5 ± 0.4	*NA*	2.5 ± 0.4
Mean forearm length at birth (mm)	14.4 ± 0.9	14.8 ± 1.0	14.2 ± 0.7
Growth rate of forearm (mm.day^–^¹)²	1.2	1.0	1.2
Growth rate of body mass (g.day^–^¹)²	0.3	NA	NA
Epiphyseal gap fusion rate (mm.day^–^¹)^3^	−0.05	−0.01	−0.05
Mean percent of adult forearm length at birth (%)	32.2 ± 2.2	33.0 ± 2.7	31.8 ± 1.7
Light brown pigmentation on head	Days 4–5	–	–
Medium pigmentation on head & shoulders	Days 5–8	–	–
Dark pigmentation on head & shoulders	Days 7–9	–	–
Sparse fur emerging	From Day 8	–	–
Velvet fur	From Day 11	–	–
Eyes open	Days 7–9	–	–
Days old when no longer attached to mother in roost	15–17	–	–
Days old when volant	30–36	–	–
Mean percentage of adult FA when volant (%)	96–98	–	–
Days old when possibly weaned	45	–	–
Mean percentage of adult FA when possibly weaned (%)	99–100	–	–

Of the 110 (M = 67, F = 43) pups marked at OPNP in Nov–Dec 2018, 29% remained in the population one year after birth (M = 15, F = 20), and 6% were recaptured 28 months after birth (Mar 2021; M = 0, F = 7). This is consistent with longer term records, see additional demographic data on PIT-tagged pups at OPNP collected from 2014 to 2016 (Table S1). At the time of weaning in 2018, 12% (M = 8, F = 6) of pups at OPNP were not recaptured, however, the true number of pups who did not survive to weaning is likely lower as not all boxes were checked at this timepoint and a 2.7% rate of PIT tag loss has been reported in *C. gouldii* (van Harten et al. 2021). Pups became reproductively active in their first year of life; males had distended testes (evidence of spermatogenesis) and sperm present in their epididymis from two months of age (DE *pers. obs*.), and 11 out of 12 recaptured females in the 2019 breeding season (Nov 2019–Jan 2020) were lactating or postlactating. One female was recaptured with an unfurred pup in February 2020 after being recorded as postlactating in January, suggesting a second pregnancy in her first year.

### Timing of birth

**Fig. 2:**
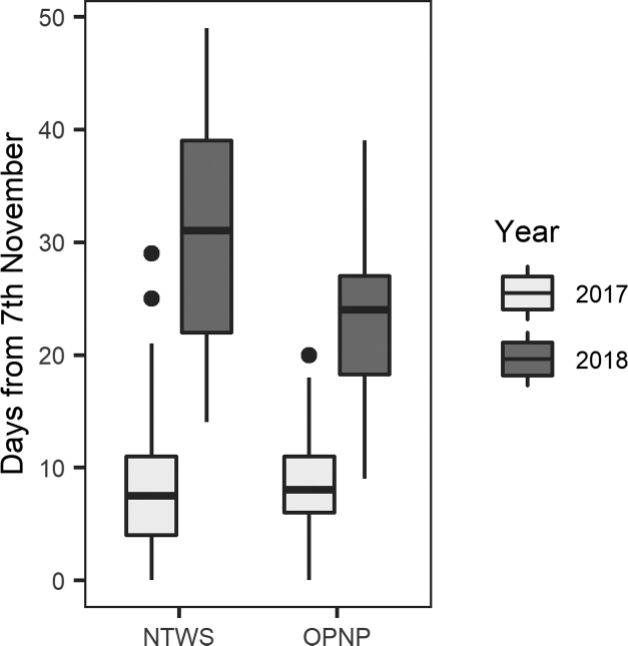
Pup date of birth (number of days from the first birth within the study [November 7th]) within each site (*n* = 256 mothers; NTWS: Nangak Tamboree Wildlife Sanctuary, *n* = 125; OPNP: Organ Pipes National Park, *n* = 131) and year. Boxes indicate the median value and first and third quartiles, whiskers extend to 1.5 times the inter-quartile range with outliers beyond.

The median parturition date in 2017 (15th November; Fig. 2) was 18 days earlier than the median parturition date in 2018 (2nd December). The spread of birth dates was also much greater in 2018 than 2017 (Fig. 2).

### Size at birth

**Fig. 3:**
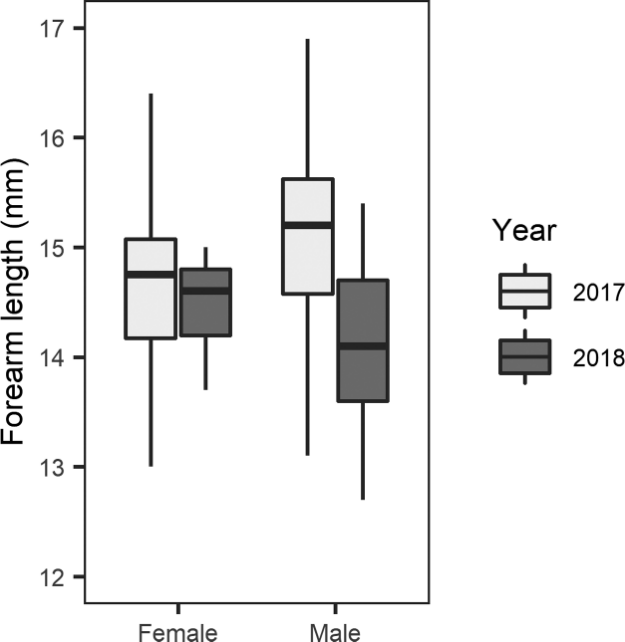
Forearm lengths (mm) on day of birth in 2017 (female: *n* = 14; male: *n* = 8) and 2018 (female: *n* = 13; male: *n* = 21) *C. gouldii* neonates (*n* = 56 pups). Boxes indicate the median value and first and third quartiles, whiskers extend to 1.5 times the inter-quartile range with outliers beyond.

The mean (± SD) forearm length of neonates captured on day 1 was 14.4 ± 1.0 mm (range between 12.7 mm and 16.9 mm; 32.2 ± 2.2% of mother's forearm length). Neonates in 2017 had significantly longer forearms at birth than in 2018 (2017 = 14.8 ± 0.9 mm, 2018 = 14.2 ± 0.7 mm; F = 4.43, *P* < 0.05, Table S2, Fig. 3). Forearm length did not differ significantly between sexes (F = 14.6 ± 0.7 mm, M = 14.4 ± 1.0 mm; *P* > 0.05, Table S2, Fig. 3) and the effects of year did not differ between sexes (*P* > 0.05, Table S2).

Body mass on day 1 ranged from 2.0–3.2 g (mean 2.4 ± 0.2 g), with each pup averaging 9–15% of mother's body weight. Length of the epiphyseal gap at birth ranged from 2.0–3.1 mm (mean 2.7 ± 0.04 mm). There was no difference in epiphyseal gap length at birth between years or sexes (all *P* > 0.05).

### Postnatal growth

**Fig. 4:**
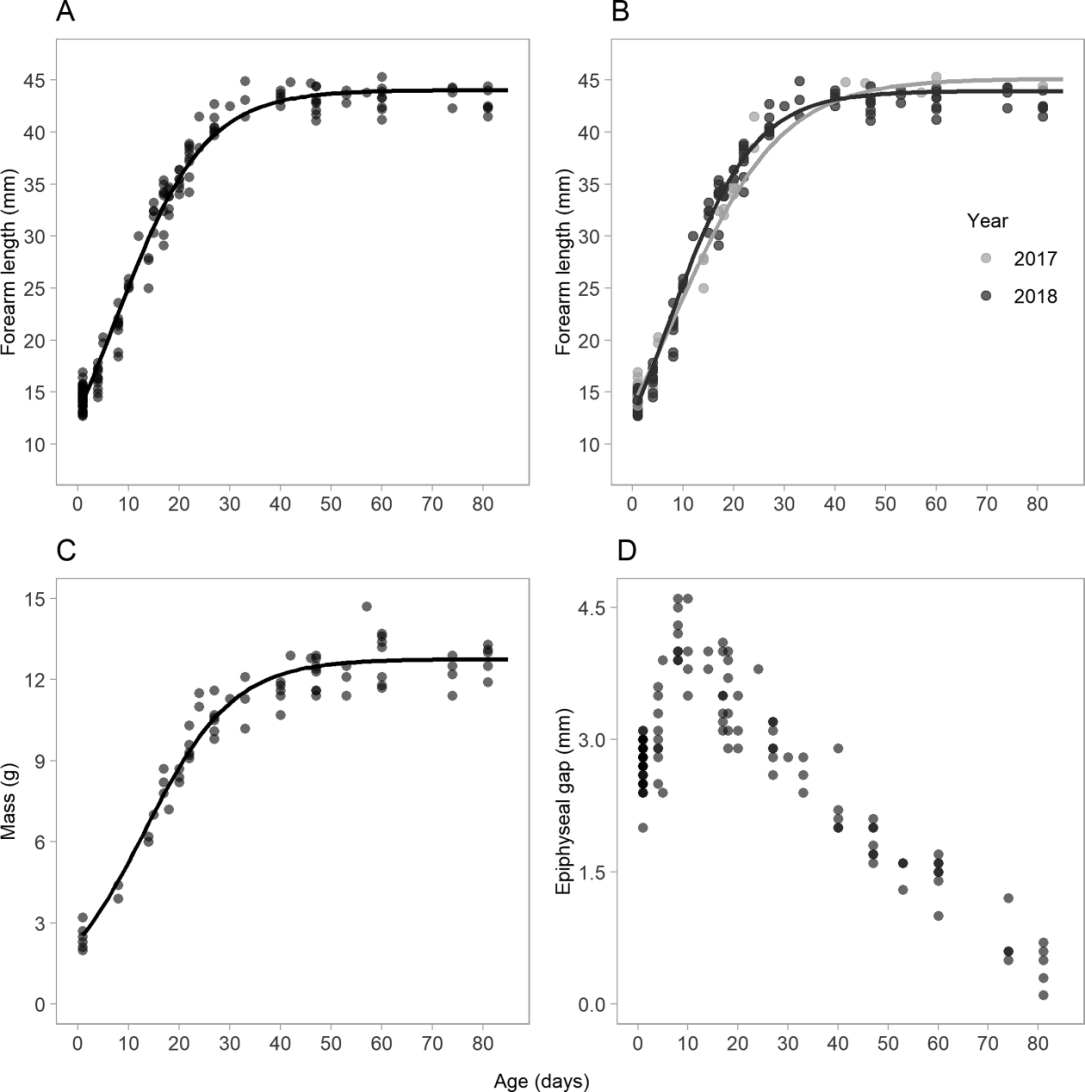
Empirical growth curves for (A) forearm length (mm), (B) forearm length split by year, (C) body mass (g), and (D) length of fourth metacarpal epiphyseal gap (mm) in *C. gouldii* (*n* = 56 pups) from day 1 to 81. Dotted line represents logistic equation for forearm length and mass.

The logistic model was the most parsimonious of the three non-linear growth models fitted to forearm length and body mass (Table 2; Fig. 4A and C). The asymptotic value for forearm length and mass were estimated to be 44.02 ± 0.14 mm and 12.75 ± 0.22 g, respectively (Table 2). Allowing differences between years resulted in a more parsimonious model of forearm length growth. Forearm lengths grew faster and plateaued earlier in 2018 than in 2017, although forearm lengths in 2017 grew to a larger average length (Table 2, Fig. 4B). Forearm length more than doubled in the first 3 weeks to an average of 35.8 mm (Fig. 4A). The epiphyseal gap increased in size until day 10, then decreased until fusion between day 80–90 (Fig. 4D).

**Table 2:** Parameters obtained from growth curves of forearm length (mm) and body mass (g) in *C. gouldii*. All growth models included Maternal ID as a random effect. Abbreviations: y = body mass or forearm length at age (t), A = asymptotic value , β = displacement on x-axis, and k = growth rate constant (days^−1^), AIC = Akaike's Information Criterion, BIC = Bayesian Information Criterion. *P* < 0.001 for all data.

			Forearm length		
		Body mass	(mm) ± SE		
Model	Parameter	(g) ± SE	*Combined*	*2017*	*2018*
**Logistic**	A	12.75 ± 0.22	44.02 ± 0.14	45.11 ± 0.55	43.93 ± 0.60
y(t) = A/{1+βe^–kt^}	β	4.49 ± 0.38	2.37 ± 0.04	2.27 ± 0.06	2.09 ± 0.07
	k	0.11 ± 0.01	0.11 ± 0.00	0.09 ± 0.00	0.06 ± 0.00
	AIC	134.14	742.05	672.11	
**Gompertz**	A	13.09 ± 0.25	44.28 ± 0.21		
y(t) = A exp{-βe^–kt^}	β	1.86 ± 0.08	1.25 ± 0.01		
	k	0.08 ± 0.01	0.09 ± 0.00		
	AIC	136.02	754.84		
**Von Bertalanffy**	A	NA^1^	44.38 ± 0.22		
y(t) = {A^1- β^-θe^–kt^}^1/(1-β)^	β	NA	0.35 ± 0.00		
	k	NA	0.08 ± 0.00		
	AIC	NA	780.64		

1 Insufficient data to produce Von Bertalanffy model for mass.

### Age estimation

**Fig. 5:**
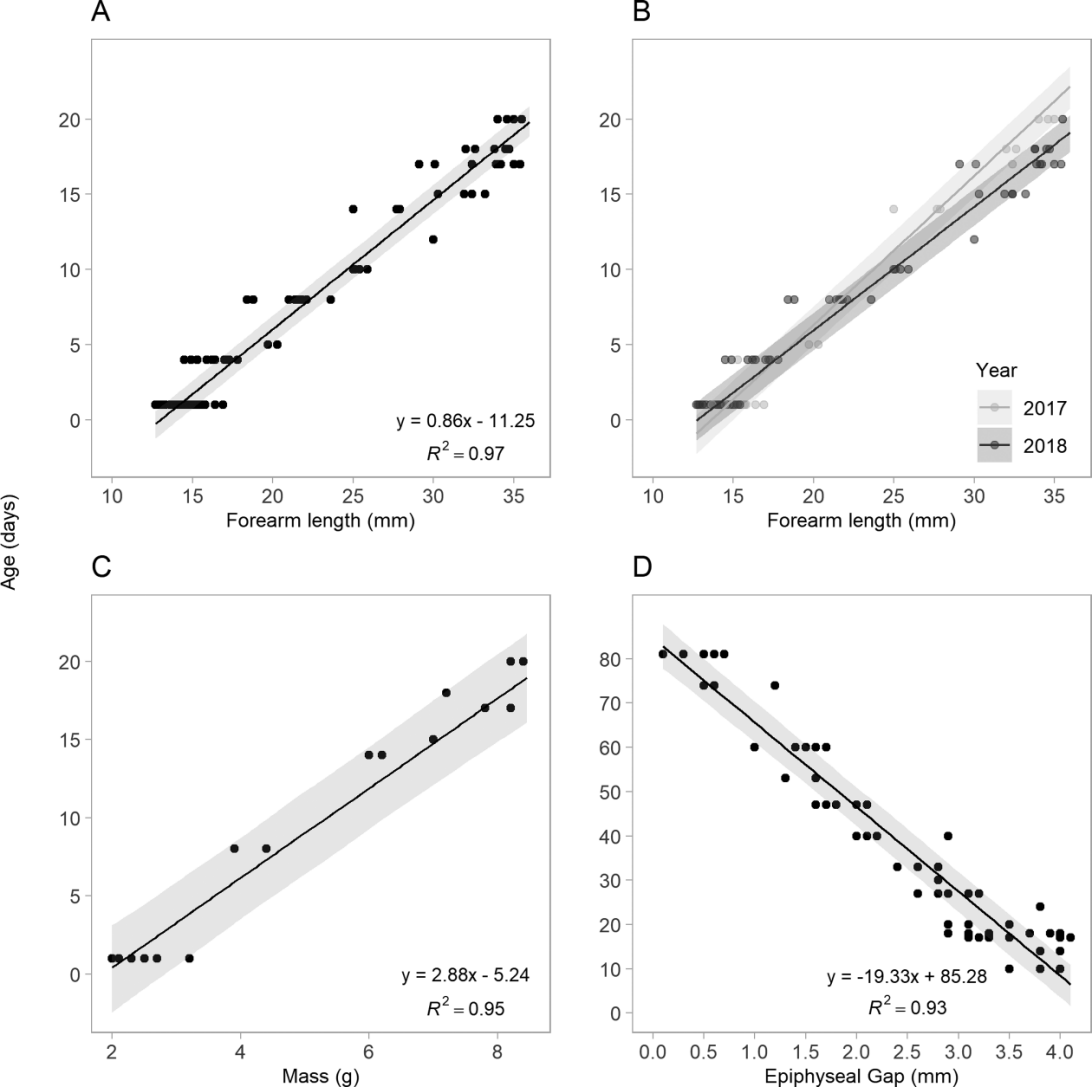
The early linear portion of postnatal growth used to estimate age in (A) forearm length (1–21 days), (B) forearm length separated by year (1–21 days), (C) body mass (1–21 days), (D) epiphyseal gap (10–81 days) in juvenile *C. gouldii* (*n* = 56 pups). Confidence intervals (grey shading; CI) were calculated for agepredictive equations using bootstrapping with 500 samples.

The equation for age estimation based on forearm length is valid when forearm length is ≤ 36.3 mm (Fig. 5A) and the equation for body mass is valid when mass is ≤ 8.8 g (Fig. 5C). The epiphyseal gap length displayed a linear increase from days 1–9, and a linear decrease from day 10 to 80. The age prediction of *C. gouldii* based on length of epiphyseal gap is restricted to post-day 10 measurements, when forearm lengths are ≥ 24.5 mm (Fig. 5D).

Bats born in 2018 had shorter forearms at birth but grew significantly faster over the first 20 days than bats born in 2017 (*t* = −2.09, *P* < 0.05; Fig. 5B). Faster growth in 2018 reflect significant interactions between day and year, with the difference in mean forearm length between years increasing from day 7 to 20 (day × year: *t* = 7.55, *P* < 0.01). There was no difference in the rate of epiphyseal gap fusion between years, or between sexes (*P* > 0.05).

### Fluctuations in body mass throughout first year of life

**Fig. 6:**
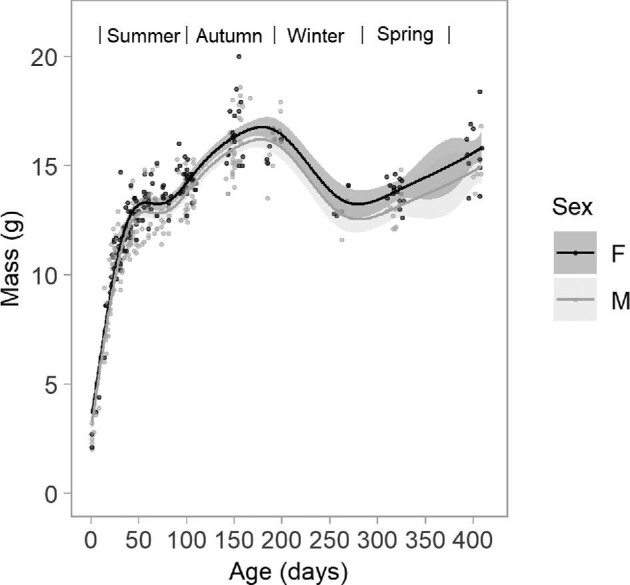
Fluctuation in body mass (g) in female (F) and male (M) *C. gouldii* across first year of life (*n* = 136 pups). Line represents generalized additive model (GAM). Approximate 95% CI were defined as mean body mass estimates plus or minus two times the standard error of these estimates.

Females were on average heavier (0.5 ± 0.1 g) than males across the first year of life from day 50 onwards (*t* = −4.5, *P* > 0.05, Fig. 6). Body mass for both sexes plateaued around day 45 until late summer, then peaked in late Autumn. Mass declined over winter, reaching the lowest point at the end of winter before increasing in early spring.

### Climatic variability between years

**Fig. 7:**
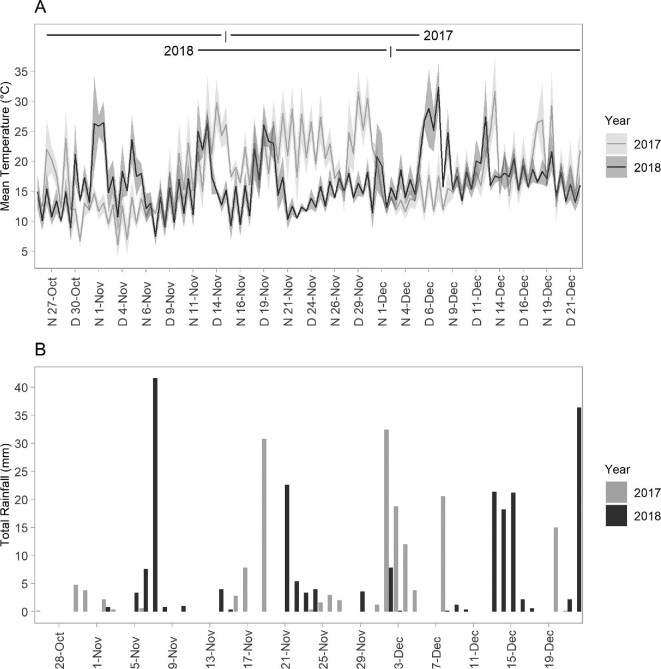
Mean daily (D) and nightly (N) temperatures (°C) and total daily rainfall (mm) for the timeframe encompassing 20 days pre- and post-median birth date in 2017 and 2018 at Organ Pipes National Park. Shading around mean daily and nightly temperatures represents ± 1 standard deviation of the mean. The vertical black line represents the median date of birth for 2017 (15th Nov) and 2018 (2nd Dec), and the horizontal black lines indicate 20 days pre- and post-birth (2017: 26th Oct–5th Dec; 2018: 12th Nov–22nd Dec).

November (late-gestation) daily minimum and maximum temperatures differed significantly between years (minimum: *t* = −2.19, *P* < 0.05; maximum: *t* = −4.40, *P* < 0.05; Table S3 & S4). The mean daily and nightly temperatures in 2017 were higher on most days than 2018 during late gestation (from 12–30 November; Fig. 7). Patterns in mean daily and nightly temperatures were similar between the two sites (Figure S1). Temperature and rainfall across early- and mid-gestation were relatively similar across years (Aug–Oct; Fig. S1A–D). Total rainfall was higher during November 2018 (NTWS: 114.6 mm, OPNP: 98.6 mm; Fig. 7) than November 2017 (NTWS: 44.6 mm, OPNP: 51.6 mm, Fig. 7A).

## Discussion

Our results provide the first postnatal growth models for an Australian insectivorous microbat, *C. gouldii*, a species with a wide distribution across much of the continent. Together, these equations predict the age of *C. gouldii* pups based on the capture of individuals from birth up to 80 days of age. Postnatal growth patterns to estimate age of young or calculate weaning dates are valuable tools for other researchers in behavioral, physiological, and ecological studies (Kunz and Hood 2000). Furthermore, collecting detailed life history data is an important first step before we can make predictions about effects of anthropogenic climate change on wildlife.

Our results show there was decreased reproductive synchrony in 2018 (births were spread over more days), and low levels of reproductive seasonality across the two years of the study (median birth date was 18 days later in 2018). At our study sites, November (late-gestation) 2018 was cooler and wetter than November 2017, so our results are consistent with past observations of links between cool and wet weather during pregnancy and delayed parturition and decreased prenatal growth in bats (Racey and Swift 1981; Hood et al. 2002; Willis et al. 2006). Temperate-zone bats tend to have highly seasonally synchronized reproduction (Tuttle and Stevenson 1982), and hence are influenced by changing weather patterns. Cool ambient temperatures and rain reduce insect abundance (Williams 1961; Taylor 1963), and during these weather conditions bats may use torpor to conserve energy (Willis and Cooper 2009). However, torpor delays parturition and prenatal development (Willis et al. 2006). In response to cooler temperatures and higher rainfall during November 2018, *C. gouldii* may have utilized torpor to conserve energy and delay parturition (Racey and Swift 1981; Ransome and McOwat 1994; Willis et al. 2006), which may have consequences for survival of juveniles (Frick et al. 2010; Barclay 2012). Potential links between weather and postnatal growth and survival would likely be exacerbated by climate change, which is predicted to increase the frequency of extreme weather events (Stott 2016).

*Chalinolobus gouldii* pups were born smaller in 2018 but grew at a faster rate during the initial 20-day growth period. As described above, small birth size has been linked to cool and wet weather in late pregnancy (Hood et al. 2002). Evidence for climatic conditions influencing postnatal growth has been reported in bat species, however, these show faster growth rates are linked to warm temperatures and/or dry weather (Koehler and Barclay 2000; Reiter 2004; Eghbali and Sharifi 2019) with the exception of *Tadarida brasiliensis* (Allen et al. 2010). However, there does not appear to be any major differences in climate during the growth period of our two years. It is possible that delayed parturition results in postpartum compensatory growth, whereby pups increase their postnatal growth rate to avoid reaching a small adult size. This may increase the animal's reproductive fitness but may come at a cost to the animal's health later in life (Metcalfe and Monaghan 2001; Hector and Nakagawa 2012). Compensatory growth has been recorded in other bat species (Tumlison 1990; Lin et al. 2011); notably Hoying and Kunz (1998) found compensatory growth in both mass and forearm length in *Pipistrellus subfalvus* pups after a period of cold weather.

Interannual differences in *C. gouldii* reproduction could be due to site differences, rather than climatic conditions *per se*, due to the confounding effect of site and year in our study design. For example, unmeasured factors such as landscape structure and insect availability may influence reproduction in *C. gouldii*. Previous studies provide evidence for increased diurnal roost temperatures altering reproductive physiology, resulting in differences in timing of parturition, size at birth (Hood et al. 2002; Willis et al. 2006), and postnatal growth (Hoying and Kunz 1998; Reiter 2004; Allen et al. 2010). There are fewer mature hollow-bearing trees at OPNP, compared to NTWS, due to more recent revegetation at this reserve (Griffiths et al. 2020). Consequently, bats at OPNP were always present in the boxes, while the NTWS bats would also utilize alternate roosts, most likely hollows in large, old trees (Evans and Lumsden 2011). Timber or plywood bat boxes do not mimic the thermal properties of natural tree hollows, with artificial roosts reaching substantially hotter diurnal temperatures and cooler night-time temperatures than ambient (Griffiths et al. 2018). Thus, slower postnatal growth rates at the NTWS may be a consequence of more stable thermal microclimates within tree hollows. Furthermore, maternal colonies may choose to roost in artificial structures with more variable roost microclimates, as the higher roost temperatures could provide energy savings while sustaining lactation (Speakman and Racey 1987; Law and Chidel 2007). However, this can be a risk if these artificial roosts reach upper thermal tolerance limits and there have been accounts of mass bat deaths in plywood bat boxes on hot days (Flaquer et al. 2014; Griffiths 2021). Unfortunately, it is difficult to monitor bats inhabiting natural tree roosts, which poses problems for studies investigating intraspecific variation in pup size at birth and postnatal growth between bats in artificial roosts compared to natural tree roosts. However, it is plausible that increased temperatures in artificial roosts may cause faster postnatal growth rates, and thus pups are weaned earlier allowing for a second reproductive attempt, as occurred in our box-roosting *C. gouldii*. It is unknown whether a second pregnancy occurs in *C. gouldii* that primarily roost in natural tree hollows.

The present study found no sex differences in forearm lengths of *C gouldii* on the day of birth, during the growth period, or when they reached full-sized adult dimensions. We did, however, find sex differences in body mass fluctuations across the first year of life, with females being on average heavier at all timepoints after the initial growth period. This is consistent with findings in *C. gouldii* at another location in suburban Melbourne (Dixon and Huxley 1989) and in the Mallee region of Victoria (Lumsden and Bennett 1995). The heavier adult weight but not adult size of females is likely a consequence of requiring high fat reserves to support pregnancy and lactation, particularly since conception occurs in late winter (August) when food resources are still scarce. Reverse sexual dimorphism is present in other vespertilionid bat species, albeit the females are often larger in size (i.e., FA length) as well as heavier (Reynolds 1999; Hood et al. 2002). In addition, both sexes of *C gouldii* appear to be reproductively active in their first year.

Our results suggest that changes in weather during the breeding season may alter *C. gouldii* parturition dates, size at birth, and postnatal growth. Given predictions of increased temperatures and reduced rainfall across the Victorian range of *C. gouldii* (DELWP 2020), climate change may be associated with earlier pup births and possibly faster postnatal growth, although this may also coincide with lower insect abundance and different insect composition due to range and demographic shifts from altered insect phenology (Marshall et al. 2020). Future studies could test reproduction in *C. gouldii* in different climate zones of Australia, and extend the models developed in the current study as a powerful means of understanding climate influences on microbat reproduction and postnatal growth.

## Supplementary Material

obac030_Supplemental_Files

## Data Availability

The data underlying this article and analysis code are available in Open Science Framework, at https://osf.io/w5cxm/?view_only=9bea5060d8954f0593bb94dfec070d4a.
