# Supplementary material for: Size at Birth, Postnatal Growth, and Reproductive Timing in an Australian Microbat
Source: Integr Org Biol. 2022 Jul 29;4(1):obac030. doi: 10.1093/iob/obac030 (PMC9436771; doi:10.1093/iob/obac030)
Supplement: obac030_Supplemental_Files [file obac030_supplemental_files.zip › Table S1.docx]

| **Year** |  | **TOTAL** | **Not captured at weaning^1^** | | **Gone before April** | | **Present April/June** | | **Present Aug** | | **Present Oct** | | **Present Dec** | | **Stayed > Dec** | | **TOTAL** | |
| --- | --- | --- | --- | --- | --- | --- | --- | --- | --- | --- | --- | --- | --- | --- | --- | --- | --- | --- |
|  |  |  | **M** | **F** | **M** | **F** | **M** | **F** | **M** | **F** | **M** | **F** | **M** | **F** | **M** | **F** | **M** | **F** |
| **2014** | **N** | **104** | **-** | **-** | 30 | 19 | 11 | 4 | 1 | 3 | 4 | 2 | 0 | 2 | 7 | 21 | **53** | **51** |
|  | **%** |  | **-** | **-** | 28.8 | 18.3 | 10.6 | 3.8 | 1.0 | 2.9 | 3.8 | 1.9 | 0.0 | 1.9 | 6.7 | 20.2 | **51.0** | **49.0** |
| **2015** | **N** | **167** | **-** | **-** | 54 | 30 | 20 | 5 | 1 | 3 | 4 | 1 | 6 | 10 | 8 | 25 | **93** | **74** |
|  | **%** |  | **-** | **-** | 32.3 | 18.0 | 12.0 | 3.0 | 0.6 | 1.8 | 2.4 | 0.6 | 3.6 | 6.0 | 4.8 | 15.0 | **55.7** | **44.3** |
| **2016** | **N** | **194** | **-** | **-** | 60 | 45 | 24 | 20 | 2 | 3 | 2 | 5 | 1 | 3 | 9 | 20 | **98** | **96** |
|  | **%** |  | **-** | **-** | 30.9 | 23.2 | 12.4 | 10.3 | 1.0 | 1.5 | 1.0 | 2.6 | 0.5 | 1.5 | 4.6 | 10.3 | **50.5** | **49.5** |
| **2018** | **N** | **110** | 8 | 6 | 27 | 11 | 17 | 6 |  |  | 6 | 5 | NA^2^ | NA^2^ | 9 | 15 | **67** | **43** |
|  | **%** |  | 7.3 | 5.5 | 24.5 | 10.0 | 15.5 | 5.5 | 0.0 | 0.0 | 5.5 | 4.5 | NA | NA | 8.2 | 13.6 | **60.9** | **39.1** |

^1^ Not all boxes were checked at weaning during 2018.

^2^ No data was collected in Dec 2019.
