# Supplementary material for: Size at Birth, Postnatal Growth, and Reproductive Timing in an Australian Microbat
Source: Integr Org Biol. 2022 Jul 29;4(1):obac030. doi: 10.1093/iob/obac030 (PMC9436771; doi:10.1093/iob/obac030)
Supplement: obac030_Supplemental_Files [file obac030_supplemental_files.zip › Table S2.docx]

| **Model** | **Sum Sq** | **Mean Sq** | **NumDF** | **DenDF** | **F value** | **Pr(>F)** |
| --- | --- | --- | --- | --- | --- | --- |
| *fa ~ year*sex + (1 \| mum id)* |  |  |  |  |  |  |
| year | 0.83 | 0.83 | 1 | 28.90 | 4.57 | < 0.05 |
| sex | 0.00 | 0.00 | 1 | 33.30 | 0.00 | 0.95 |
| year:sex | 0.58 | 0.58 | 1 | 33.30 | 3.18 | 0.08 |
