# Supplementary material for: Size at Birth, Postnatal Growth, and Reproductive Timing in an Australian Microbat
Source: Integr Org Biol. 2022 Jul 29;4(1):obac030. doi: 10.1093/iob/obac030 (PMC9436771; doi:10.1093/iob/obac030)
Supplement: obac030_Supplemental_Files [file obac030_supplemental_files.zip › Table S3.docx]

|  | **Estimate** | **Std. Error** | **t-value** | **Pr(>\|t\|)** | **Sig. level** |
| --- | --- | --- | --- | --- | --- |
| *Minimum temps* |  |  |  |  |  |
| (Intercept) | 10.51333 | 0.5987 | 17.56 | <2.00E-16 | *** |
| siteOPNP | 0.36333 | 0.84669 | 0.429 | 0.667903 |  |
| year2018 | -0.02333 | 0.84669 | -0.028 | 0.978019 |  |
| monthAUG | -4.93914 | 0.83984 | -5.881 | 5.10E-09 | *** |
| monthDEC | 3.2286 | 0.83984 | 3.844 | 0.000126 | *** |
| monthFEB | 2.68267 | 0.88802 | 3.021 | 0.002566 | ** |
| monthJAN | 4.74874 | 0.85396 | 5.561 | 3.22E-08 | *** |
| monthJUL | -5.34333 | 0.84669 | -6.311 | 3.72E-10 | *** |
| monthJUN | -5.94 | 0.84669 | -7.016 | 3.57E-12 | *** |
| monthMAR | 3.8246 | 0.85396 | 4.479 | 8.13E-06 | *** |
| monthMAY | -3.44559 | 0.83984 | -4.103 | 4.32E-05 | *** |
| monthNOV | 2.98667 | 0.84669 | 3.527 | 0.000433 | *** |
| monthOCT | -1.07785 | 0.83984 | -1.283 | 0.199566 |  |
| monthSEP | -2.89667 | 0.84669 | -3.421 | 0.000642 | *** |
| siteOPNP:year2018 | -0.54 | 1.19741 | -0.451 | 0.652078 |  |
| siteOPNP:monthAUG | -0.54075 | 1.18771 | -0.455 | 0.648972 |  |
| siteOPNP:monthDEC | -0.78914 | 1.18771 | -0.664 | 0.506531 |  |
| siteOPNP:monthFEB | -0.65933 | 1.23737 | -0.533 | 0.594221 |  |
| siteOPNP:monthJAN | -0.92863 | 1.19774 | -0.775 | 0.438284 |  |
| siteOPNP:monthJUL | -0.76559 | 1.19257 | -0.642 | 0.521 |  |
| siteOPNP:monthJUN | 0.47 | 1.19741 | 0.393 | 0.694738 |  |
| siteOPNP:monthMAR | -0.27546 | 1.19774 | -0.23 | 0.81814 |  |
| siteOPNP:monthMAY | 0.07215 | 1.18771 | 0.061 | 0.951569 |  |
| siteOPNP:monthNOV | -1.07 | 1.19741 | -0.894 | 0.371692 |  |
| siteOPNP:monthOCT | -0.93108 | 1.18771 | -0.784 | 0.433218 |  |
| siteOPNP:monthSEP | -0.97667 | 1.19741 | -0.816 | 0.414839 |  |
| year2018:monthAUG | -0.44753 | 1.19257 | -0.375 | 0.707522 |  |
| year2018:monthDEC | 0.96806 | 1.19257 | 0.812 | 0.417076 |  |
| year2018:monthFEB | 1.52733 | 1.23737 | 1.234 | 0.217284 |  |
| year2018:monthJAN | 0.62255 | 1.19774 | 0.52 | 0.603303 |  |
| year2018:monthJUL | 1.17667 | 1.19741 | 0.983 | 0.325936 |  |
| year2018:monthJUN | 0.83519 | 1.21392 | 0.688 | 0.491564 |  |
| year2018:monthMAR | -1.63073 | 1.19774 | -1.362 | 0.173575 |  |
| year2018:monthMAY | 1.71631 | 1.20326 | 1.426 | 0.153982 |  |
| year2018:monthNOV | -2.64452 | 1.20805 | -2.189 | 0.028757 | * |
| year2018:monthOCT | -0.42548 | 1.19257 | -0.357 | 0.721311 |  |
| year2018:monthSEP | -1.77905 | 1.20805 | -1.473 | 0.141069 |  |
| siteOPNP:year2018:monthAUG | 0.63989 | 1.68312 | 0.38 | 0.703867 |  |
| siteOPNP:year2018:monthDEC | 0.54043 | 1.68312 | 0.321 | 0.748191 |  |
| siteOPNP:year2018:monthFEB | 0.60029 | 1.73668 | 0.346 | 0.729657 |  |
| siteOPNP:year2018:monthJAN | 0.7311 | 1.68678 | 0.433 | 0.66477 |  |
| siteOPNP:year2018:monthJUL | 0.5157 | 1.68655 | 0.306 | 0.759824 |  |
| siteOPNP:year2018:monthJUN | -0.39852 | 1.70511 | -0.234 | 0.815236 |  |
| siteOPNP:year2018:monthMAR | 0.51987 | 1.68678 | 0.308 | 0.757976 |  |
| siteOPNP:year2018:monthMAY | 0.24703 | 1.6907 | 0.146 | 0.883856 |  |
| siteOPNP:year2018:monthNOV | 1.25119 | 1.70093 | 0.736 | 0.462104 |  |
| siteOPNP:year2018:monthOCT | 1.07269 | 1.68312 | 0.637 | 0.524019 |  |
| siteOPNP:year2018:monthSEP | 1.05905 | 1.70093 | 0.623 | 0.533631 |  |
|  |  |  |  |  |  |
| *Maximum temps* |  |  |  |  |  |
|  | Estimate | Std. Error | t-value | t value | Pr(>\|t\|) |
| (Intercept) | 20.98333 | 0.77198 | 27.181 | <2.00E-16 | *** |
| siteOPNP | -0.40333 | 1.09175 | -0.369 | 0.711858 |  |
| year2018 | 2.33 | 1.09175 | 2.134 | 0.033001 | * |
| monthAUG | -6.48333 | 1.08291 | -5.987 | 2.71E-09 | *** |
| monthDEC | 5.31344 | 1.08291 | 4.907 | 1.03E-06 | *** |
| monthFEB | 5.95595 | 1.11107 | 5.361 | 9.69E-08 | *** |
| monthJAN | 7.15 | 1.09175 | 6.549 | 8.10E-11 | *** |
| monthJUL | -6.53817 | 1.08291 | -6.038 | 2.00E-09 | *** |
| monthJUN | -6.60667 | 1.09175 | -6.051 | 1.84E-09 | *** |
| monthMAR | 7.57473 | 1.08291 | 6.995 | 4.10E-12 | *** |
| monthMAY | -4.01559 | 1.08291 | -3.708 | 0.000217 | *** |
| monthNOV | 6.25 | 1.09175 | 5.725 | 1.27E-08 | *** |
| monthOCT | 0.61667 | 1.08291 | 0.569 | 0.56914 |  |
| monthSEP | -3.42 | 1.09175 | -3.133 | 0.001769 | ** |
| siteOPNP:year2018 | 0.01333 | 1.54397 | 0.009 | 0.993111 |  |
| siteOPNP:monthAUG | 0.13237 | 1.53147 | 0.086 | 0.931136 |  |
| siteOPNP:monthDEC | -0.3386 | 1.53147 | -0.221 | 0.825049 |  |
| siteOPNP:monthFEB | 0.14619 | 1.5713 | 0.093 | 0.925887 |  |
| siteOPNP:monthJAN | -0.12677 | 1.53773 | -0.082 | 0.934307 |  |
| siteOPNP:monthJUL | -0.07086 | 1.53147 | -0.046 | 0.963102 |  |
| siteOPNP:monthJUN | 0.35667 | 1.54397 | 0.231 | 0.817343 |  |
| siteOPNP:monthMAR | -0.39344 | 1.53147 | -0.257 | 0.79729 |  |
| siteOPNP:monthMAY | 0.03237 | 1.53147 | 0.021 | 0.983142 |  |
| siteOPNP:monthNOV | -0.22333 | 1.54397 | -0.145 | 0.885009 |  |
| siteOPNP:monthOCT | 0.3872 | 1.53147 | 0.253 | 0.800435 |  |
| siteOPNP:monthSEP | 0.51333 | 1.54397 | 0.332 | 0.739579 |  |
| year2018:monthAUG | -1.90419 | 1.53147 | -1.243 | 0.213936 |  |
| year2018:monthDEC | -1.7429 | 1.53147 | -1.138 | 0.255289 |  |
| year2018:monthFEB | -0.58 | 1.5713 | -0.369 | 0.712093 |  |
| year2018:monthJAN | -1.40849 | 1.53773 | -0.916 | 0.359847 |  |
| year2018:monthJUL | -2.29774 | 1.53147 | -1.5 | 0.133748 |  |
| year2018:monthJUN | -2.47095 | 1.55769 | -1.586 | 0.112899 |  |
| year2018:monthMAR | -4.64613 | 1.53147 | -3.034 | 0.00246 | ** |
| year2018:monthMAY | -1.72988 | 1.55151 | -1.115 | 0.265055 |  |
| year2018:monthNOV | -6.82195 | 1.55061 | -4.4 | 1.17E-05 | *** |
| year2018:monthOCT | -1.70241 | 1.5444 | -1.102 | 0.270514 |  |
| year2018:monthSEP | -2.57548 | 1.55769 | -1.653 | 0.098475 | . |
| siteOPNP:year2018:monthAUG | 0.13505 | 2.16582 | 0.062 | 0.950288 |  |
| siteOPNP:year2018:monthDEC | -0.08753 | 2.16582 | -0.04 | 0.96777 |  |
| siteOPNP:year2018:monthFEB | -0.57762 | 2.22215 | -0.26 | 0.79495 |  |
| siteOPNP:year2018:monthJAN | -0.28 | 2.17025 | -0.129 | 0.897363 |  |
| siteOPNP:year2018:monthJUL | 0.11892 | 2.16582 | 0.055 | 0.956218 |  |
| siteOPNP:year2018:monthJUN | -0.28238 | 2.19323 | -0.129 | 0.897573 |  |
| siteOPNP:year2018:monthMAR | 0.38344 | 2.16582 | 0.177 | 0.859501 |  |
| siteOPNP:year2018:monthMAY | -0.01345 | 2.18004 | -0.006 | 0.995079 |  |
| siteOPNP:year2018:monthNOV | 0.33529 | 2.1882 | 0.153 | 0.878243 |  |
| siteOPNP:year2018:monthOCT | -0.46673 | 2.17498 | -0.215 | 0.830119 |  |
| siteOPNP:year2018:monthSEP | -0.01786 | 2.19323 | -0.008 | 0.993505 |  |
