# Supplementary material for: Size at Birth, Postnatal Growth, and Reproductive Timing in an Australian Microbat
Source: Integr Org Biol. 2022 Jul 29;4(1):obac030. doi: 10.1093/iob/obac030 (PMC9436771; doi:10.1093/iob/obac030)
Supplement: obac030_Supplemental_Files [file obac030_supplemental_files.zip › Table S4.docx]

|  | **Jan** | **Feb** | **Mar** | **Apr** | **May** | **June** | **July** | **Aug** | **Sep** | **Oct** | **Nov** | **Dec** |
| --- | --- | --- | --- | --- | --- | --- | --- | --- | --- | --- | --- | --- |
| **Mean Min Temp** |  |  |  |  |  |  |  |  |  |  |  |  |
| *NTWS* |  |  |  |  |  |  |  |  |  |  |  |  |
| 2017 | 15.3 ± 0.7 | 13.2 ± 0.6 | 14.3 ± 0.6 | 10.5 ± 0.6 | 7.1 ± 0.5 | 4.6 ± 0.3 | 5.2 ± 0.7 | 5.6 ± 0.6 | 7.6 ± 0.6 | 9.4 ± 0.7 | 13.5 ± 0.8 | 13.7 ± 0.6 |
| 2018 | 15.9 ± 0.7 | 14.7 ± 0.6 | 12.7 ± 0.5 | 10.5 ± 0.6 | 8.8 ± 0.5 | 5.4 ± 0.6 | 6.3 ± 0.6 | 5.1 ± 0.6 | 5.8± 0.7 | 9.0 ± 0.7 | 10.8 ± 0.7 | 14.7 ± 0.6 |
| *OPNP* |  |  |  |  |  |  |  |  |  |  |  |  |
| 2017 | 14.7 ± 0.6 | 12.9 ± 0.6 | 14.4 ± 0.6 | 10.9 ± 0.5 | 7.5 ± 0.5 | 5.4 ± 0.3 | 4.8 ± 0.5 | 5.4 ± 0.5 | 7.0 ± 0.6 | 8.9 ± 0.6 | 12.8 ± 0.8 | 13.3 ± 0.6 |
| 2018 | 15.5 ± 0.7 | 14.5 ± 0.7 | 12.8 ± 0.6 | 10.3 ± 0.5 | 8.9 ± 0.5 | 5.3 ± 0.5 | 5.3 ± 0.5 | 5.0 ± 0.5 | 5.7 ± 0.4 | 9.0 ± 0.7 | 10.8 ± 0.7 | 14.3 ± 0.6 |
| **Mean Max temp** |  |  |  |  |  |  |  |  |  |  |  |  |
| *NTWS* |  |  |  |  |  |  |  |  |  |  |  |  |
| 2017 | 28.1 ± 1.0 | 26.9 ± 1.1 | 28.6 ± 0.7 | 21.0 ± 0.7 | 17.0 ± 0.4 | 14.4 ± 0.3 | 14.4 ± 0.3 | 14.5 ± 0.5 | 17.6 ± 0.7 | 21.6 ± 0.8 | 27.2 ± 1.3 | 26.3 ± 1.3 |
| 2018 | 29.1 ± 1.1 | 28.7 ± 0.7 | 26.2 ± 0.8 | 23.3 ± 0.7 | 17.6 ± 0.6 | 14.2 ± 0.4 | 14.5 ± 0.4 | 14.9 ± 0.4 | 17.3± 0.6 | 22.0 ± 0.6 | 22.2 ± 0.6 | 26.9 ± 1.0 |
| *OPNP* |  |  |  |  |  |  |  |  |  |  |  |  |
| 2017 | 27.6 ± 1.0 | 26.7 ± 1.1 | 27.8 ± 0.8 | 20.6 ± 0.8 | 16.6 ± 0.4 | 14.3 ± 0.3 | 14.0 ± 0.3 | 14.2 ± 0.5 | 17.7 ± 0.7 | 21.6 ± 0.8 | 26.6 ± 1.2 | 25.6 ± 1.0 |
| 2018 | 28.3 ± 1.2 | 27.9 ± 0.8 | 25.8 ± 0.8 | 22.9 ± 0.8 | 17.2 ± 0.6 | 13.9 ± 0.3 | 14.1 ± 0.4 | 14.8 ± 0.3 | 17.4 ± 0.6 | 21.8 ± 0.7 | 22.5 ± 1.0 | 26.1 ± 1.0 |
