# Supplementary figures and images for: Size at Birth, Postnatal Growth, and Reproductive Timing in an Australian Microbat
Source: Integr Org Biol. 2022 Jul 29;4(1):obac030. doi: 10.1093/iob/obac030 (PMC9436771; doi:10.1093/iob/obac030)

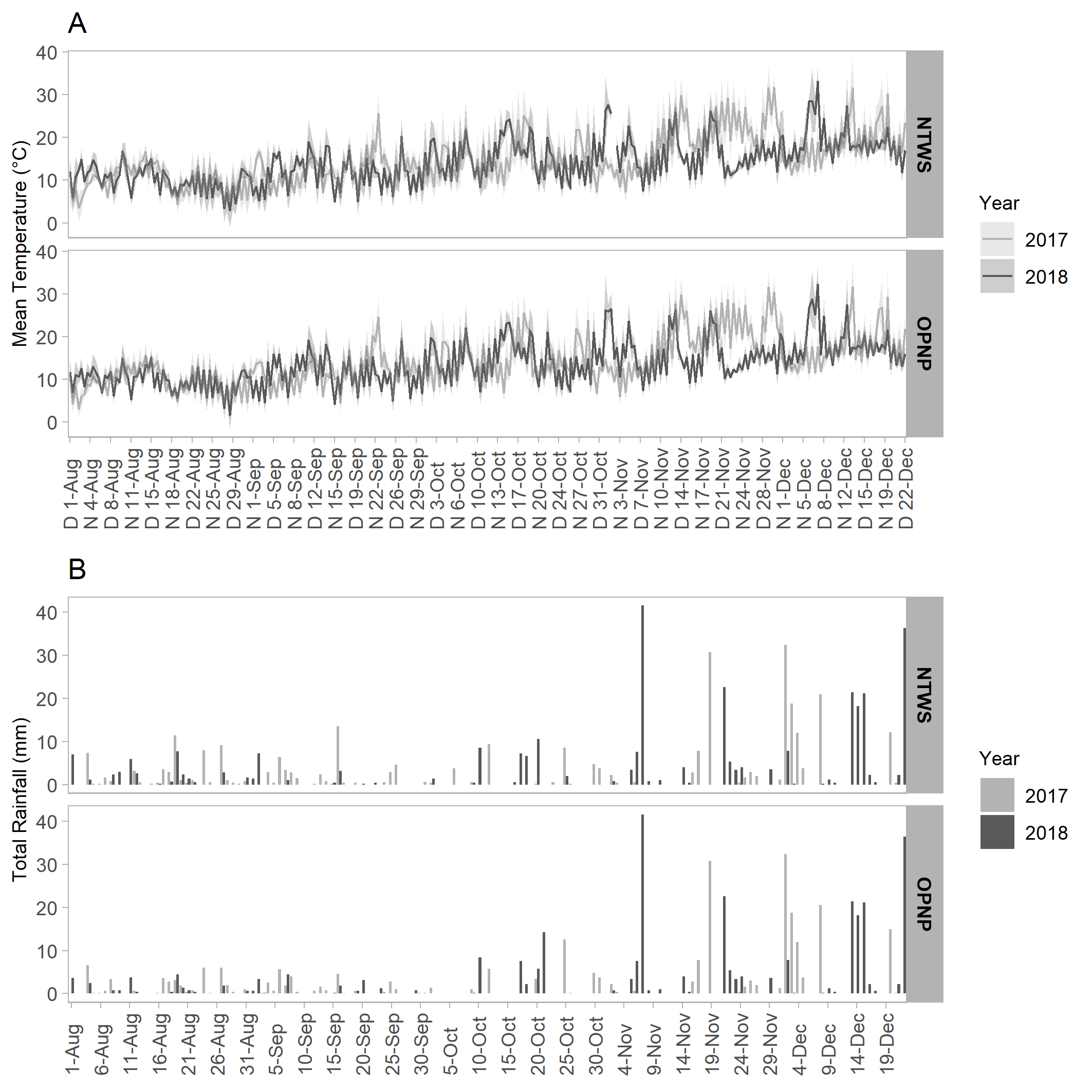

Supplement: obac030_Supplemental_Files [file obac030_supplemental_files.zip › Figure S1.tiff]
